# Multi-omics analysis reveals the molecular regulatory network underlying the prevention of *Lactiplantibacillus plantarum* against LPS-induced salpingitis in laying hens

**DOI:** 10.1186/s40104-023-00937-x

**Published:** 2023-11-17

**Authors:** Dan Song, Aike Li, Bingxu Chen, Jia Feng, Tao Duan, Junlin Cheng, Lixian Chen, Weiwei Wang, Yuna Min

**Affiliations:** 1 College of Animal Science and Technology, Northwest A&F University, Shaanxi, People’s Republic of China; 2 Key Laboratory of Grain and Oil Biotechnology of National Food and Strategic Reserves Administration, Academy of National Food and Strategic Reserves Administration, Beijing, People’s Republic of China

**Keywords:** Laying hen, Metabolome, Microbiome, Microencapsulated *Lactiplantibacillus plantarum*, Salpingitis, Transcriptome

## Abstract

**Background:**

Salpingitis is one of the common diseases in laying hen production, which greatly decreases the economic outcome of laying hen farming. *Lactiplantibacillus plantarum* was effective in preventing local or systemic inflammation, however rare studies were reported on its prevention against salpingitis. This study aimed to investigate the preventive molecular regulatory network of microencapsulated *Lactiplantibacillus plantarum* (MLP) against salpingitis through multi-omics analysis, including microbiome, transcriptome and metabolome analyses.

**Results:**

The results revealed that supplementation of MLP in diet significantly alleviated the inflammation and atrophy of uterus caused by lipopolysaccharide (LPS) in hens (*P* < 0.05). The concentrations of plasma IL-2 and IL-10 in hens of MLP-LPS group were higher than those in hens of LPS-stimulation group (CN-LPS group) (*P* < 0.05). The expression levels of *TLR2*, *MYD88*, *NF-κB*, *COX2*, and *TNF-α* were significantly decreased in the hens fed diet supplemented with MLP and suffered with LPS stimulation (MLP-LPS group) compared with those in the hens of CN-LPS group (*P* < 0.05). Differentially expressed genes (DEGs) induced by  MLP were involved in inflammation, reproduction, and calcium ion transport. At the genus level, the MLP supplementation significantly increased  the abundance of *Phascolarctobacterium*, whereas decreased the abundance of *Candidatus_Saccharimonas* in LPS challenged hens (*P* < 0.05). The metabolites altered by dietary supplementation with MLP were mainly involved in galactose, uronic acid, histidine, pyruvate and primary bile acid metabolism. Dietary supplementation with MLP inversely regulates LPS-induced differential metabolites such as LysoPA (24:0/0:0) (*P* < 0.05).

**Conclusions:**

In summary, dietary supplementation with microencapsulated *Lactiplantibacillus plantarum* prevented salpingitis by modulating the abundances of *Candidatus_Saccharimonas*, *Phascolarctobacterium*, *Ruminococcus_torques_group* and *Eubacterium_hallii_group* while downregulating the levels of plasma metabolites, *p*-tolyl sulfate, *o*-cresol and *N*-acetylhistamine and upregulating *S*-lactoylglutathione, simultaneously increasing the expressions of *CPNE4, CNTN3* and *ACAN* genes in the uterus, and ultimately inhibiting oviducal inflammation.

**Supplementary Information:**

The online version contains supplementary material available at 10.1186/s40104-023-00937-x.

## Background

According to the findings of the Food and Agriculture Organization (FAO) in 2021, poultry accounts for 38.6% of the overall global meat consumption. In terms of egg production, the global output reached 86.3878 million tons in the same year, with China alone contributing 34.4% to worldwide production [1]. It is projected that in the forthcoming decade, poultry consumption will surpass half of the global meat consumption growth [2]. Demand for poultry products have been shifting gradually from quantity to quality [3]. The use of antibiotics is restricted in laying hens because of their possible residues in eggs. Meanwhile, the lack of antibiotics in laying hens increases the risk of bacterial inflammation such as salpingitis, which brings down the laying performance and egg quality [4]. Salpingitis is a common and frequent disease in laying hens and is characterized by distension and inflammation of the oviduct and caseous exudates [5], which also is an infection of the oviduct by bacteria from the intestinal lumen and respiratory tract, resulting in 10% to 25% of the elimination rate in chickens [6]. And the formation and coloring of eggshells were directly affected by inflammation in the uterus of the oviduct. This disease led to decreased animal welfare, considerable economic losses, and the risk of horizontal and vertical transmission of pathogenic *E. coli* [7]. Consequently, preventing salpingitis through appropriate nutritional regulation is an urgent need in poultry production. Probiotics is the commonly used feed additive for nutritional regulation against inflammation, in which *Lactiplantibacillus plantarum* is widely used and taken as the antibiotic alternative [8].

Dietary supplementation with *Lactiplantibacillus plantarum* increased laying rate, improved intestinal microbiota composition [9], enhanced intestinal barrier and immune function, inhibited apoptosis [8, 10], and reduced ammonia emissions [11]. *Lactiplantibacillus plantarum* regulated the intensity of inflammatory response by enhancing mucosal barrier function and phagocytosis ability of phagocytes, stimulating immune cells to produce anti-inflammatory cytokines and antibodies, and inhibiting the secretion of pro-inflammatory cytokines [12–14]. Research had shown that *Lactiplantibacillus plantarum* could effectively prevent *E. coli* infection, regulated intestinal immune function, and effectively treated intestinal infections caused by *E. coli* [15]. Cao et al. [16] demonstrated that *Lactiplantibacillus plantarum* 1.2567 suppressed necrotizing enteritis by regulating intestinal mucosal immune response, improving villus structure and antioxidant defense system in broilers, and achieved anti-inflammatory effects by inhibiting neutrophil recruitment and reducing the release of inflammatory mediators.

Host-microbe interactions are closely related to health and disease. The key mechanism underlying these interactions is that metabolites derived from the microbiota not only provide the host with nutrients, but also involve in the regulation of immune development. However, it remains unclear whether *Lactiplantibacillus plantarum* prevents salpingitis and whether it is correlated with the altered blood metabolites derived from gut microbiome. Salpingitis of laying hens is mainly induced by *E. coli*, in which lipopolysaccharide (LPS) is its principal pathogenic component [17]. Lipopolysaccharide, a prominent bioactive constituent of the cell wall of Gram-negative bacteria, is known to play a pivotal role in triggering immune responses, acute inflammation, and tissue damage, and is commonly used to induce local or systemic inflammation models [18]. We invented a salpingitis model as a patent using LPS from *E. coli* O55:B5 in hens [19]. This study aims to elucidate the multi-omics molecular regulatory network for prevention of microencapsulated *Lactiplantibacillus plantarum* (MLP) against LPS-induced salpingitis through microbiome, metabolome and transcriptome analyses.

## Materials and methods

### Animal ethics

All experimental procedures were approved by the Animal Care and Use Committee of Northwest A&F University, Yangling, China.

### Experimental materials

The MLP (1.0 × 10^10^ CFU/g) was prepared by our patented emulsion technology (ZL202011208280.7). This technique used multilayer coating to enhance the stress resistance of *Lactiplantibacillus plantarum* [20]. Lipopolysaccharide obtained from *E. coli* O55:B5 was purchased from Sigma Aldrich (Shanghai, China).

### Experimental design and sample collection

A total of 270 34-week-old healthy Hy-Line Brown laying hens were randomly divided into 3 groups with 6 replicates in each group and 15 birds in each replicate for 24 weeks. The hens in the control group (CN) and LPS-stimulation group (CN-LPS) were fed a basal diet, while the hens in the MLP with LPS-stimulation group (MLP-LPS) was given a diet containing 0.1% MLP. After a 24-week feeding period, a total of 3 birds were selected from each replicate (*n* = 18). Empty capsules and capsules containing LPS at 2 mg/kg body weight were placed into the oviducts of the hens in the control (CN) and experimental groups (CN-LPS and MLP-LPS), respectively.

For the collection of blood from wing veins, one laying hen was randomly selected from each replicate after the 12 h LPS challenge. Blood samples were centrifuged for 10 min at 4 °C at 3,000 r/min and then stored at −20 °C. The laying hens were exsanguinated by inhalation anesthesia using carbon dioxide (CO_2_) after blood sample collection, and the oviduct was dissected immediately. In addition, a piece of uterus measuring approximately 1 cm^3^ was fixed in formalin buffer and another piece was frozen immediately in liquid nitrogen at −80 °C for future RNA-seq analysis. In the concurrent phase, fresh cecal contents were also collected for further microbiota analysis [21].

### Diets and management

The basal diet was prepared based on NRC (1994) [22]. The composition and nutrient levels of the basal diet were shown in Table S1, Additional file 1. It was provided with artificial light for 16 h/d and routine immunizations for the hens in a three-story stepped cage, 3 hens per cage. It was kept at a temperature of 21–24 °C. During the entire experimental period, diets and water were available ad libitum.

### Uterine histological assessment

After more than 24 h fixing of uterine tissue in 4% formalin buffer, the tissue samples were embedded in paraffin, sectioned, and stained with hematoxylin eosin (HE). After staining with HE, the sections of tissue samples were examined under bright field light microscope (BX43, Olympus, Tokyo, Japan) at a magnification of 200 for histopathological changes [23, 24]. In this study, the scoring criteria for pathology were modified from those originally published by Liu et al. [25]. Pathological symptoms were classified into two categories: glandular atrophy and interstitial inflammation. Each symptom was scored separately, and the sum of the scores for each symptom was the total pathological score of the tissue. The pathological scoring criteria for glandular atrophy were as follows: 0 was normal and 1–4 points represented the percentage of < 25%, 25%–50%, 50%–75% and > 75% for the atrophied mucosa of the entire lamina propria, respectively. The criteria for scoring interstitial inflammation pathologically were as follows: 0 indicated normal, 1 indicated mild cell infiltration, 2 indicated mild cell infiltration with inflammatory lesions and capillary congestion, and 3 indicated moderate cell infiltration with inflammatory lesions and capillary congestion.

### Measurement of serum cytokine concentrations

The concentrations of cytokines (IL-2, interleukin-2, Cat#: MK5249A; IL-6, interleukin-6, Cat#: MK5250A; IL-10, interleukin-10, Cat#: MK5023A; INF-γ, interferon-γ, Cat#: MK2660A) were determined using chicken-specific ELISA kits (Beijing Zhongke Kaiser Technology Co., Ltd., China).

### Real-time PCR

The protocol for real-time PCR analysis was conducted according to the previous study [26]. Total RNA extraction from uterus was performed using the RNA Easy Fast Tissue/Cell kit DP451 (Tiangen Biochemical Technology Co., Ltd., Beijing, China), OD_260/__280_ and OD_260/__230_ ratios were used to determine the quality of the RNA [21]. Thereafter, cDNA was synthesized by PrimeScript™ RT reagent kit (TaKaRa, Dalian, China). Real-time PCR was performed according to the TB Green® Premix Ex Taq™ kit (TaKaRa, Dalian, China). Results of RT-qPCR were calculated through 2^−ΔΔCt^ method to quantify relative gene expression [27]. The sequences of primers used for real-time PCR were shown in Table S2, Additional file 1.

### Transcriptome data analysis

Transcriptome analysis included RNA extraction, library preparation, and Illumina Nova seq 6000 sequencing, read mapping and differential expression analysis and functional enrichment. Using FASTQ, the raw sequencing read data were transformed into quality checks [21]. The filtered reads were compared to the reference genome (Gallus_gallus, GCF_016700215.1) using HISAT2 software. To identify differentially expressed genes (DEGs), DESeq2 was applied with the parameters |log_2_FC| ≥ 1 and *P* value < 0.05 [28]. The R language ggplots2 was employed to map the volcanoes. After finishing DEG analysis, Gene Ontology (GO) functional enrichment as well as Kyoto Encyclopedia of Genes and Genomes (KEGG) pathway analysis were performed using Goatools (https://github.com/tanghaibao/Goatools) and KOBAS (http://kobas.cbi.pku.edu.cn/home.do). In addition, the *P*-value was calculated by hypergeometric distribution method with significant enrichment criterion *P*-value < 0.05 [29]. Finally, cluster analysis was performed on the selected differentially expressed genes.

### Gut microbiome analysis

16S rRNA high-throughput sequencing methods included DNA extraction, PCR amplification, fluorescence quantification, Illumina library construction, and sequencing [30]. For sequencing, a MiSeq PE300 platform from Illumina was used (Shanghai Majorbio Bio-Pharm Technology Co., Ltd., China). Data analysis was carried out on the Megisense cloud computing platform (https://cloud.majorbio.com). The alpha diversity indices, including Shannon, Simpson, Chao1 and ACE, were calculated with Mothur 1.30.1 using OTU information. The linear discriminant analysis (LDA) effect size (LEfSe) was calculated to identify the significantly abundant taxa (phylum to genera) of bacteria among different groups (LDA score > 2, *P* < 0.05) [31].

### Metabolomic profiling of plasma

Analysis of non-targeted metabolomics by ultra-performance liquid chromatography-tandem time of flight mass spectrometry (UPLC/Q-TOF–MS/MS; Shanghai Majorbio Bio-pharm Technology Co., Ltd., China). A 100-μL liquid sample was extracted using a 1:1 solution of methanol–acetonitrile. LC–MS analysis was performed using UHPLC-Q Exactive HF-X system (Thermo Fisher Scientific), according to Li et al. [30]. Data were pre-processed and analyzed on Megisense's cloud computing platform (http://cloud.majorbio.com/). PLS-DA (partial least squares-discriminant analysis) was performed by R package ropls (Version 1.6.2). Based on Student's *t*-test (VIP-Pred-OPLS-DA > 1, *P* < 0.05) and Kruskal–Wallis H test (multiple group comparisons), differential metabolites were screened. The final screened differential metabolites were used to build a metabolic set on which VIP value analysis and KEGG enrichment were performed.

### Statistical analysis

The data were analyzed using SPSS 20.0 (SPSS Inc., Chicago, IL, USA). Duncan's multiple comparison test was used to compare the differences between experimental groups to determine the effects of dietary treatment on the measured variables. The probability value of *P* < 0.05 was considered to be statistically significant. The data were expressed as means and pooled SEM, and graphs were generated by G*raphPad Prism 8.0 software.* The correlation analysis was conducted using Pearson's correlation coefficient.

## Results

### Pathological evaluation of uterus and inflammatory cytokines in plasma and expression of immune-related genes of uterus

**Fig. 1:**
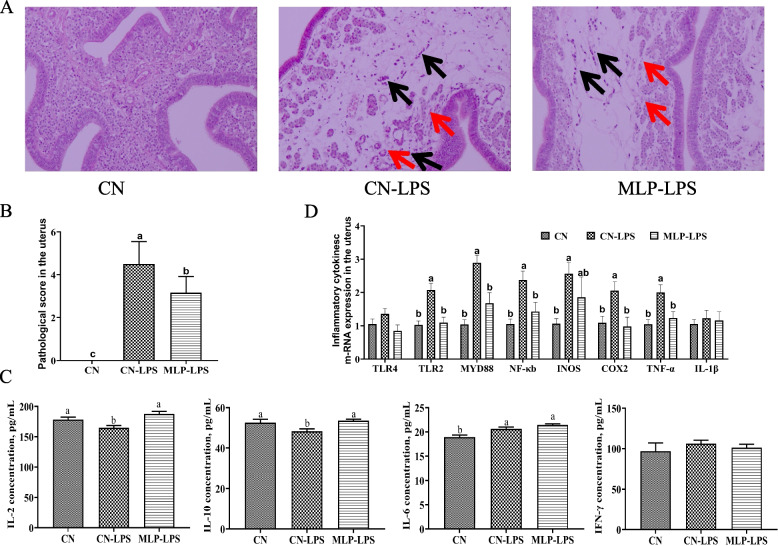
Effects of MLP on pathological evaluation and inflammatory response of laying hens challenged with LPS. **A** Pathological evaluation of uterus. Hematoxylin and erosion (H&E) staining, 200× magnification (*n* = 6). Black arrows indicate inflammation and red arrows indicate atrophy of the gland. **B** Comparison of pathological analysis score of uterus. **C** Plasma inflammatory cytokines. **D** The expression of immune-related genes of uterus. Values were means and standard errors, different letters denote significant differences among different groups (*P* < 0.05). CN, Basal diet; CN-LPS, Basal diet + LPS; MLP-LPS, Basal diet + MLP + LPS

HE staining was used to observe pathological lesions of the uterus. In the CN-LPS group, multifocal inflammatory lesions were present in the interstitium of the lamina propria and the mucosal folds of the uterus showed partial atrophy. The atrophic area accounted for 25%—50% of the total lamina propria area. According to pathological score and histological analysis, the inflammation and atrophy were relieved after the addition of MLP, and the atrophic area accounted for less than 25% of the total lamina propria area (Fig. 1A and B). Compared with the control group (CN), the plasma concentration of IL-6 was significantly increased and the plasma concentrations of IL-2 and IL-10 were decreased after LPS stimulation (*P* < 0.05). The plasma concentrations of IL-2 and IL-10 in MLP-LPS group were higher than those in CN-LPS group (*P* < 0.05). Dietary supplementation with MLP and LPS stimulation did not significantly affect the concentration of IFN-γ (*P* > 0.05) (Fig. 1C). As depicted in Fig. 1D, the expression levels of *TLR2*, *INOS*, *COX2*, *MYD88*, *NF-κB* and *TNF-α* were higher than those in CN-LPS group (*P* < 0.05). The expression levels of *TLR2*, *MYD88*, *NF-κB*, *COX2*, and *TNF-α* were significantly decreased in the MLP-LPS group, compared with CN-LPS group (*P* < 0.05).

### Transcriptome analysis

**Fig. 2:**
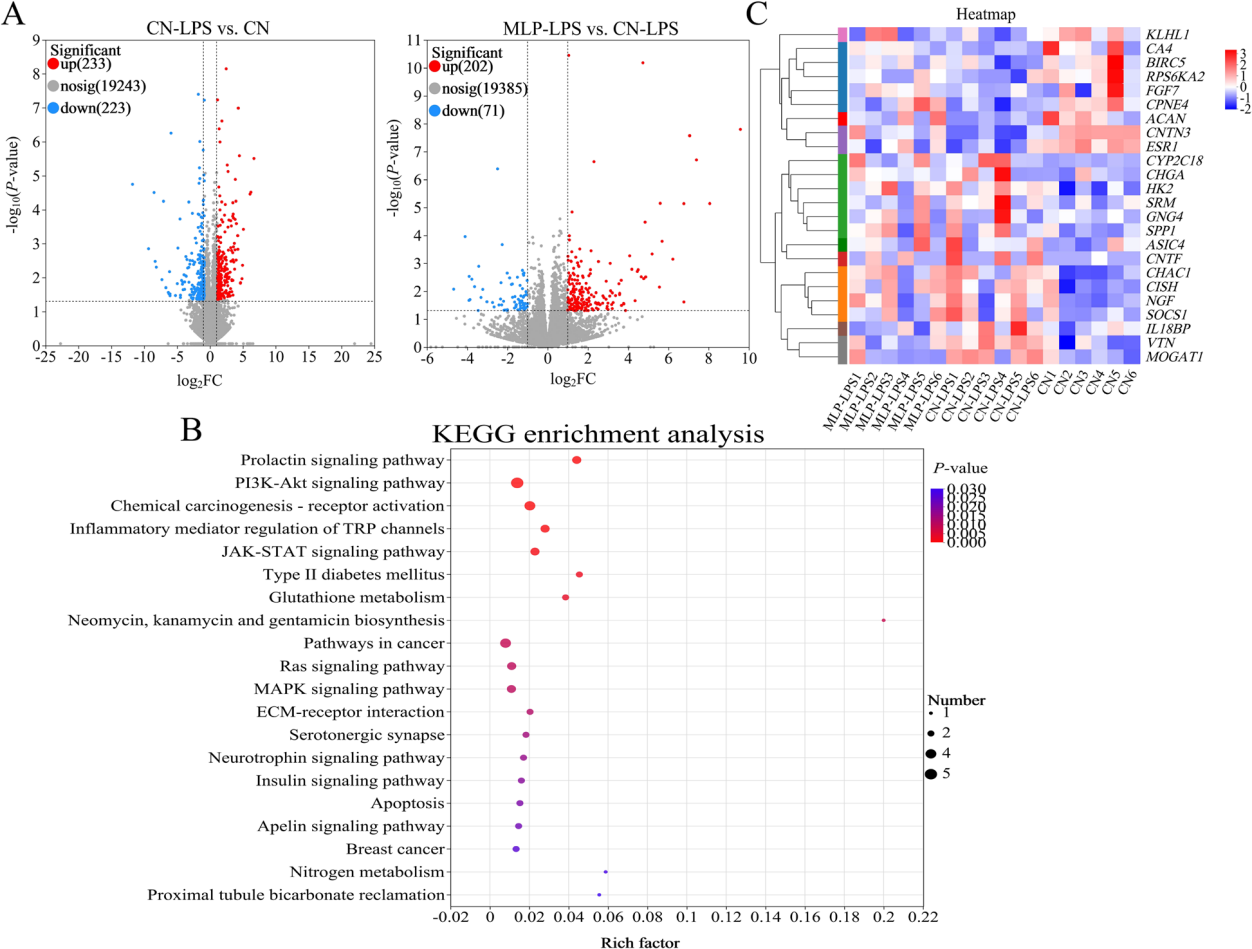
Analysis of uterine transcriptome data of laying hens stimulated with LPS by dietary MLP. **A** Volcano plot of differentially expressed genes (DEGs) in the uterus of laying hens. **B** KEGG signaling pathways enriched by DEGs. The size of the dots indicates the number of DEGs, and the color of the dots represents the *P*-value. **C** The heatmap of DEGs on CN, CN-LPS, and MLP-LPS groups, where the red indicates up-regulated genes, and the blue indicates down-regulated genes. CN, Basal diet; CN-LPS, Basal diet + LPS; MLP-LPS, Basal diet + MLP + LPS

Based on a screening for differentially expressed genes using *P* < 0.05, FC ≥ 2 and FC ≤ 0.5, there were 233 up-regulated and 233 down-regulated DEGs in the CN-LPS groups compared to the CN group (Fig. 2A). In comparison to the CN-LPS group, 202 up-regulated and 71 down-regulated DEGs were detected in the MLP-LPS group (Fig. 2A). Further screening of the DEGs by comparing the three groups resulted in the identification of 24 genes that were subjected to KEGG enrichment analysis (Fig. 2B). It revealed that the most enriched pathways among the DEGs included PI3K-Akt signaling pathway, chemical carcinogenesis-receptor activation, inflammatory mediator regulation of TRP channels, and MAPK signaling pathway. All the pathways were related with inflammation. In addition, the expression levels of *SPP1*, *HK2*, *CYP2C18*, *NGF*, *GNG4*, *CHGA*, *MOGAT1* and *VTN* were upregulated in the CN-LPS group compared to the CN group, while the expression levels of *ESR1*, *KLHL1*, *CA4*, *CPNE4*, *CNTN3* and *ACAN* were downregulated. In contrast to the CN-LPS group, the aforementioned genes were inversely regulated in the MLP-LPS group. (Fig. 2C).

To validate the results of transcriptome analysis, the relative gene expressions of candidate genes (*CA4*, *SPP1*, *HK2* and *SOCS1*) were assessed by quantitative real-time PCR (qRT-PCR). In comparison to the RNA-seq results, qRT-PCR results showed a similar expression pattern, indicating reliability of the transcriptome results (Fig. S1).

### Microbiological analysis

**Fig. 3:**
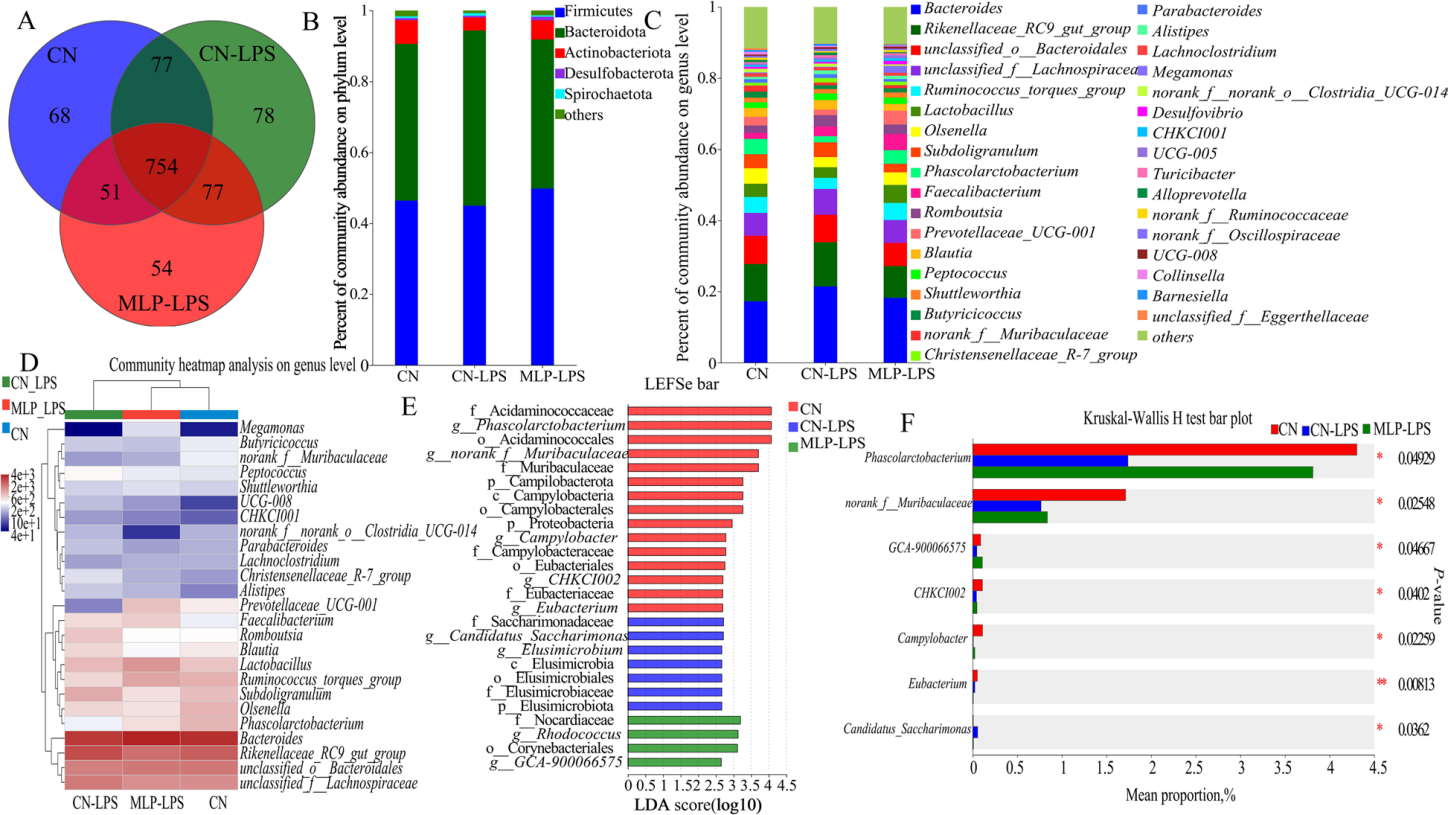
Effects of MLP on the cecal microbiota of laying hens challenged with LPS derived from *Escherichia coli*. **A** Venn diagram of operational taxonomic units (OTUs). **B** and **C** The cecal bacterial community compositions in control, LPS, and MLP groups at phylum and genus levels. The different colors of the bars represent different species, and the length of the bars represents the proportion of the species. **D** The heatmap showing the composition of the genus-level microbiota combined with the results from the cluster analysis. **E** Histogram of the LDA score reveals the most diferentially abundant taxa in CN, CN-LPS, and MLP-LPS groups. **F** Differential microorganisms were tested using the Kruskal–Wallis H test in control, LPS, and MLP groups. CN, Basal diet; CN-LPS, Basal diet + LPS; MLP-LPS, Basal diet + MLP + LPS

A total of 666,504 V3 16S rRNA amplicon sequence reads were analyzed. The maximum number of sequences per sample was 64,120 and the minimum number of sequences was 31,723. Alpha diversity analysis in Table S3 showed that the richness (ACE and Chao) and diversity (Shannon and Simpson) of cecal microflora were not significantly affected by dietary supplementation of MLP and LPS challenge (*P* > 0.05), but those in the CN-LPS group had a higher abundance than the other groups. The Venn diagram showed that all three groups shared 754 common OTUs (Fig. 3A). Subsequently, the composition of the cecal microbiota was analyzed at the phylum and genus levels. Bacteroidota and Firmicutes were the dominant phyla in each group.The abundance of Bacteroidota was higher and that of Actinobacteriota was lower in the CN-LPS group than those in the other treatment groups, while the abundance of Firmicutes in the CN-MLP group was higher than that in the CN-LPS and CN groups (Fig. 3B). At the genus level, the abundances of *Bacteroides* and *Rikenellaceae_RC9_gut_group* were higher, while the abundances of *Ruminococcus_torques_group* and *Phascolarctobacterium* were lower in the CN-LPS group than those in the CN and MLP-LPS groups. The abundances of *Lactobacillus*, *Faecalibacterium* and *Megamonas* in MLP group were higher than those in the other treatment groups (Fig. 3C and D). According to the LEfSe analysis, the microbiota of the control group was mainly enriched in *CHKCI002*, *Eubacterium*, *Campylobacter*, *norank-f-Muribaculaceae*, and *Phascolarctobacterium*; the microbiota enriched in the CN-LPS group were *Elusimicrobium* and *Candidatus-Saccharimonas*, and those enriched in the MLP-LPS group were *GCA-900066575* and *Rhodococcus* (Fig. 3E). Comparing the three groups, it was found that the CN-LPS group significantly increased the abundance of *Candidatus-Saccharimonas* while decreasing the abundance of *Phascolarctobacterium* (*P* < 0.05). In contrast, compared to the CN-LPS group, the MLP-LPS group significantly increased the abundance of beneficial bacteria *Phascolarctobacterium*, while decreasing the abundance of *Candidatus_Saccharimonas* (*P* < 0.05) (Fig. 3F).

### Metabolome analysis

**Fig. 4:**
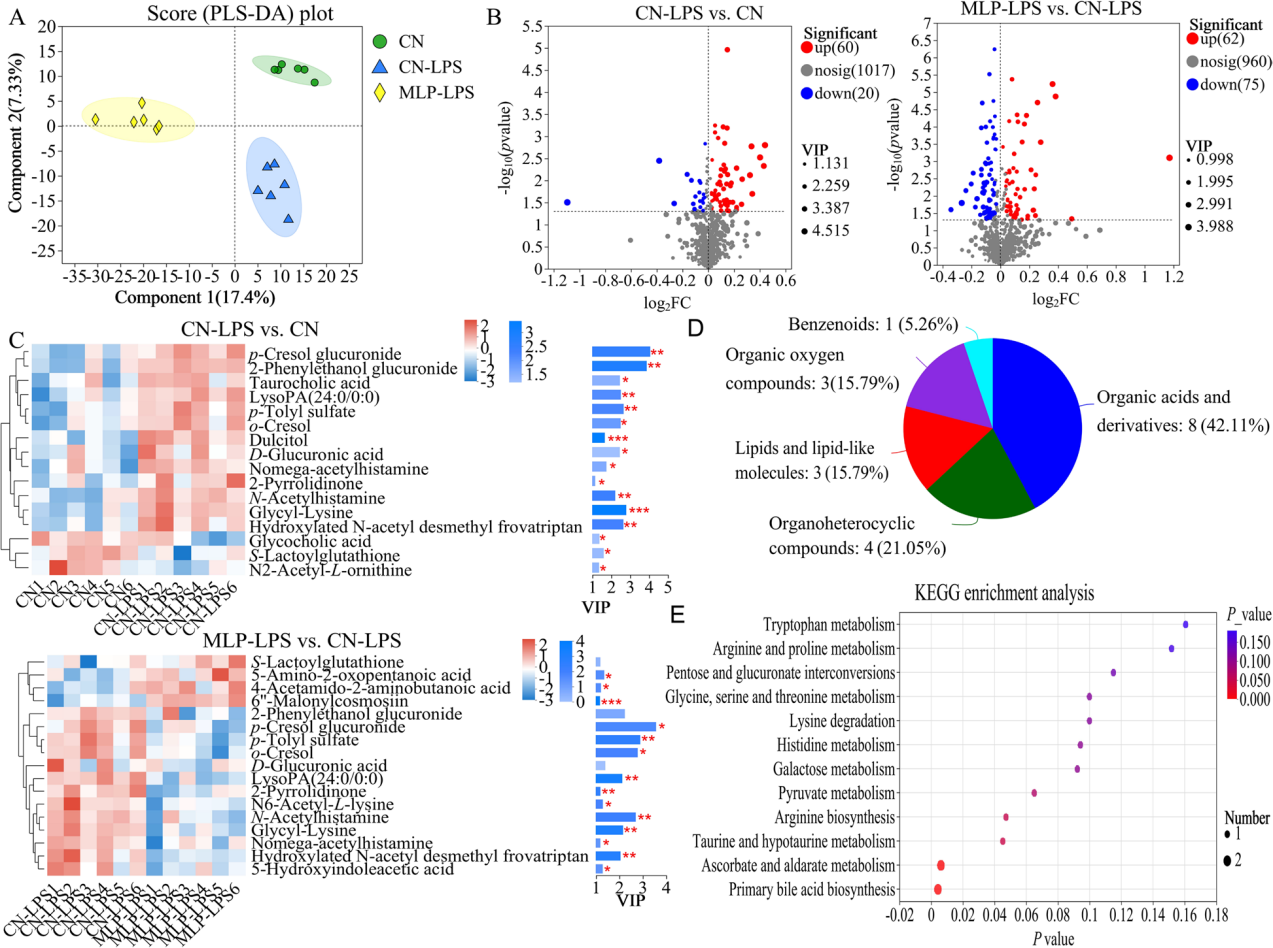
Effects of MLP on the plasma metabolites of laying hens challenged with LPS derived from *Escherichia coli*. **A** Partial least squares-discriminant analysis (PLS-DA) model of plasma metabolites. The *x*- and *y*-axes denote the first and second principal components, respectively. **B** Volcano plot of differentially plasma metabolites. **C** VIP scores analysis for differential plasma metabolites. Top 20 metabolites among VIP scores > 1 were selected and ranked based on VIP scores. The length of the bar indicates the value of the contribution of this metabolite to the difference between the two groups. The color of the bar indicates the significance of the difference between the two groups of samples. * means *P* < 0.05, ** means *P* < 0.01 and *** means *P* < 0.001. **D** Classification of differential metabolites in the HMDB database. **E** KEGG signaling pathways enriched by different metabolites (*P* < 0.05, VIP-Pred-OPLS-DA > 1). The size of the dots indicates the number of differentially abundant metabolites and the color of the dots represents the *P*-value. CN, Basal diet; CN-LPS, Basal diet + LPS; MLP-LPS, Basal diet + MLP + LPS

**Fig. 5:**
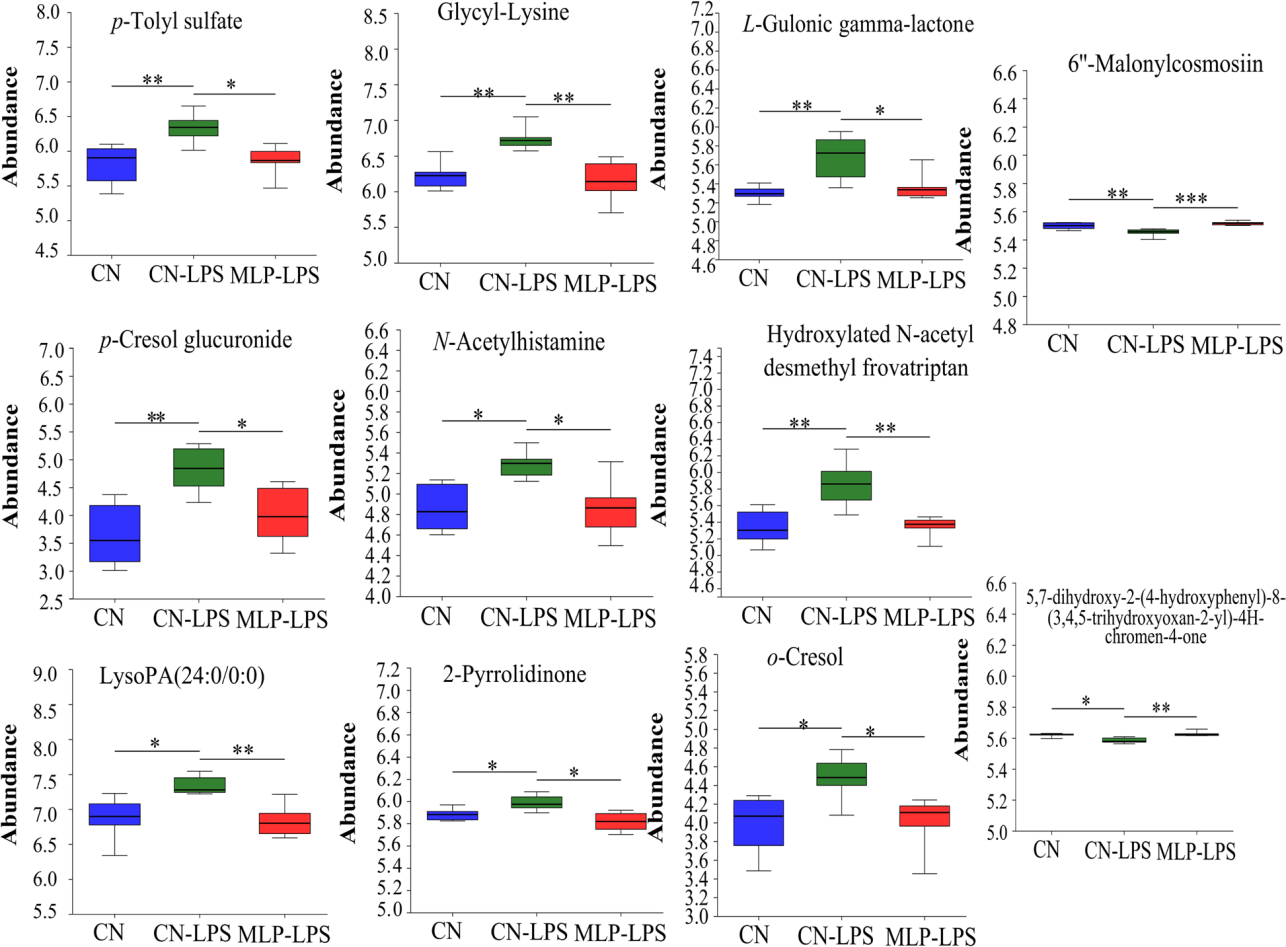
Significant changes in plasma differential metabolites were observed in the CN, CN-LPS and MLP-LPS groups. Significance was calculated by the Kruskal–Wallis H test. * means *P* < 0.05, ** means *P* < 0.01 and *** means *P* < 0.001. CN, Basal diet; CN-LPS, Basal diet + LPS; MLP-LPS, Basal diet + MLP + LPS

PLS-DA analysis revealed that metabolites in the CN, CN-LPS, and MLP-LPS groups were clearly classified into three categories (Fig. 4A). Based on the volcano plots, the CN-LPS group had 60 up-regulated and 20 down-regulated metabolites compared to the CN group. Similarly, compared with the CN-LPS group, there were 62 upregulated and 75 downregulated metabolites in the MLP-LPS group (Fig. 4B). VIP analysis of the differential metabolites revealed that *p*-cresol glucuronide had the largest difference between CN vs. CN-LPS and CN-LPS vs. MLP-LPS (Fig. 4C). After the three-group comparison, 22 differentially expressed metabolites were further screened. Among them, organic acids and derivatives accounted for 42.11% of the differential metabolites in CN, CN-LPS, and MLP-LPS, organic heterocyclic compounds accounted for 21.05%, lipids and lipid-like molecules at 15.79%, organic oxygen compounds at 15.79%, and aromatic compounds at 5.26% (Fig. 4D). KEGG enrichment analysis showed that the main enriched pathways included primary bile acid biosynthesis, galactose metabolism, ascorbate and aldarate metabolism, pyruvate metabolism and histidine metabolism (Fig. 4E). As shown in Fig. 5, compared with the CN group, the metabolites 2-pyrrolidinone, glycyl-lysine, hydroxylated *N*-acetyl desmethyl frovatriptan, *L*-gulonic gamma-lactone, LysoPA (24:0/0:0), *N*-acetylhistamine, *o*-cresol, *p*-cresol glucuronide, and *p*-tolyl sulfate were significantly upregulated (*P* < 0.05) in the CN-LPS group, while 6''-malonylcosmosiin and 5,7-dihydroxy-2-(4-hydroxyphenyl)-8-(3,4,5-trihydroxyoxan-2-yl)-4H-chromen-4-one were significantly downregulated (*P* < 0.05). The supplementation of MLP in the diet significantly alleviated the changes of metabolites induced by CN-LPS group (*P* < 0.05), with no significant difference compared to the control group (*P* > 0.05).

### Correlation analysis of microbiome, metabolome and transcriptome

**Fig. 6:**
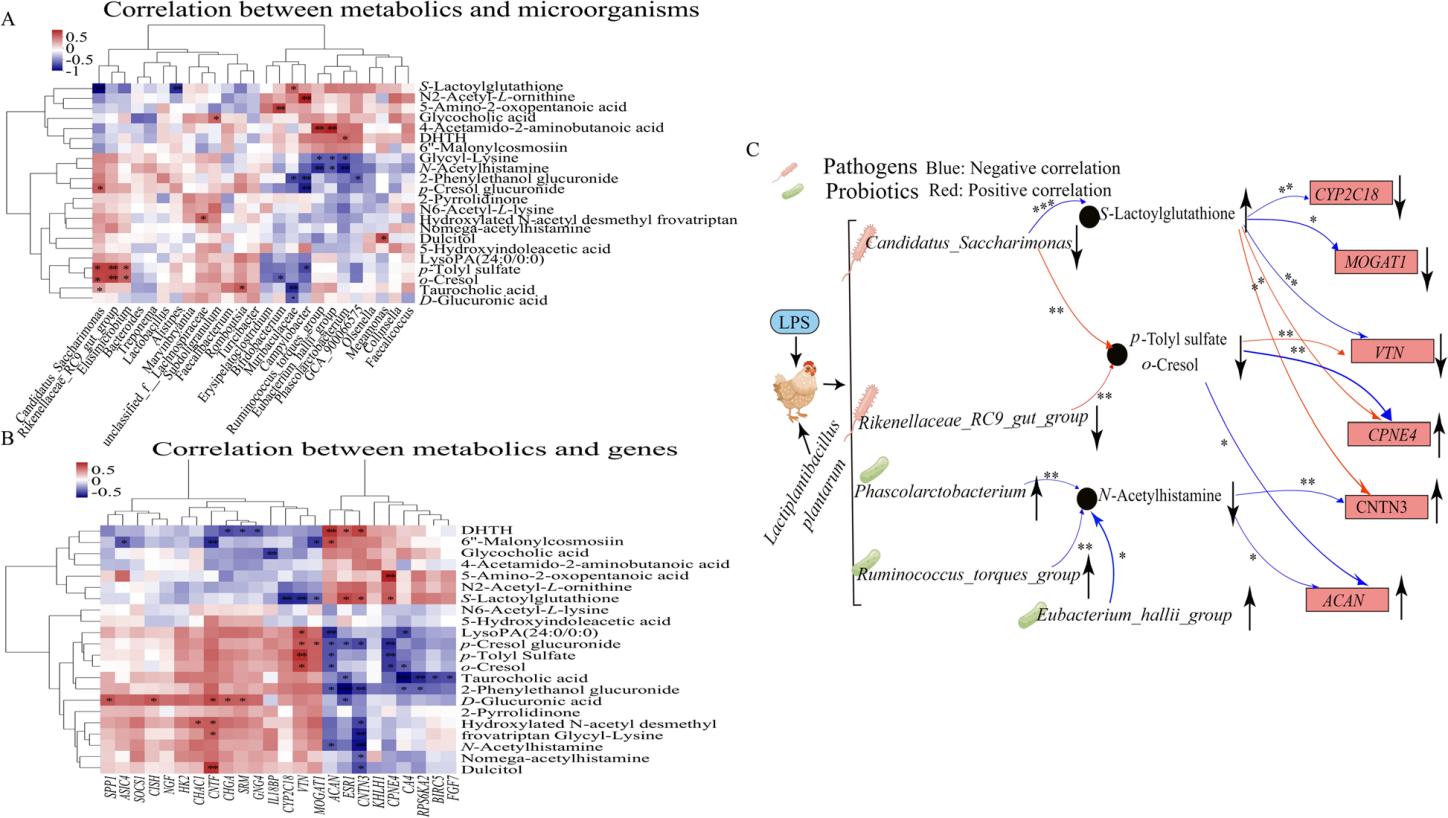
Combined multi-omics analysis. Correlation analysis between significantly differential bacteria in the cecum and differential metabolites in the plasma (**A**), significantly differential metabolites in the plasma and expressed genes (DEGs) in the uterus (**B**), and effects of MLP on microorganisms, metabolites, and genes after lipopolysaccharide stimulation (**C**). Red indicates a positive correlation; the blue indicates a negative correlation. ***, **, and * represent levels of significance (*P* < 0.001, *P* < 0.01, and *P* < 0.05, respectively). DHTH, 5,7-dihydroxy-2-(4-hydroxyphenyl)-8-(3,4,5-trihydroxyoxan-2-yl)-4H-chromen-4-one

To further analyze the preventive mechanism of MLP supplementation in preventing LPS-induced salpingitis, a correlation analysis of the microbiome, metabolome and transcriptome was performed. *S*-lactoylglutathione was significantly negatively correlated with *Candidatus_Saccharimonas*, *Alistipes*, *CYP2C18*, *VTN*, and *MOGAT1*, while it was significantly positively correlated with *Muribaculaceae*, *CNTN3*, *CPNE4*, and *ESR1*. *p*-Tolyl sulfate was significantly negatively correlated with *Campylobacter*. *CPNE4*, and *ACAN*, *o*-cresol was significantly negatively correlated with *Eubacterium_hallii_group*, *CPNE4*, *CA4*, and *ACAN*, and both *p*-tolyl sulfate and *o*-cresol were significantly positively correlated with *Candidatus_Saccharimonas*, *Rikenellaceae_RC9_gut_group*, *Elusimicrobium*, and *VTN*. N-acetylhistamine was significantly negatively correlated with *Phascolarctobacterium*, *Ruminococcus_torques_group*, *Eubacterium_hallii_group*, *CNTN3*, and *ACAN* (Fig. 6A and B).

## Discussion

Salpingitis, a common and frequent disease of laying hens, was caused by infections with biological pathogens, particularly *Escherichia coli* [32]. The purpose of this study was to investigate the molecular regulatory network of *Lactiplantibacillus plantarum* in preventing salpingitis induced by LPS derived from pathogenic *Escherichia coli*. The serious inflammation and atrophy were observed in the uterus of the oviduct after LPS stimulation. In parallel, it was observed that the levels of pro-inflammatory cytokine IL-6 were significantly elevated while the levels of anti-inflammatory cytokine IL-10 in plasma were markedly reduced. LPS-induced inflammation mainly activated the expression of genes related to MAPK/NF-κB pathway [33]. This study found that the expression levels of *TLR2*, *INOS*, *COX2*, *MYD88*, *NF-κB* and *TNF-α* were significantly increased in CN-LPS group. Recently, research had indicated that the activation of the NF-κB pathway resulted in a significant inflammatory response [34]. Dietary supplementation with MLP significantly alleviated LPS-induced inflammation and atrophy and changes in cytokine concentrations, which was consistent with Gong et al. [35] who found that *Lactiplantibacillus plantarum* improved inflammation induced by *Clostridium perfringens*. Compared with CN-LPS group, the expression levels of *TLR2*, *COX2*, *MYD88*, *NF-κB* and *TNF-α* were significantly decreased in MLP-LPS group. Zhao et al. [36] reported that *L. plantarum* induced M1 polarization and suppressed *Salmonellae*-induced inflammation by triggering TLR2/NF-κB. The inhibition by MLP against NF-κB activation may lead to lower levels of inflammatory cytokines and higher levels of anti-inflammatory cytokines, ultimately mitigating the inflammation and atrophy of oviduct caused by LPS.

To gain further insight into the mechanisms of salpingitis remission, RNA-Seq was used to assess transcriptomic changes in salpingitis alleviation in the current study. According to KEGG enrichment analysis, PI3K-Akt signaling pathway was the pathway with the most enriched genes, including *SPP1*, *VTN*, *CNG4*, *NGF*, etc. PI3K and Akt were previously shown to be involved in biological signal transduction, such as cell apoptosis [37]. Akt was the direct downstream target of PI3K. NF-κB was activated by Akt, which entered the nucleus and activated target genes to release inflammatory mediators and induce inflammatory responses [38]. CN-LPS group induced salpingitis by activating the PI3K-Akt signaling pathway in this study. This finding was consistent with that of Ni et al. [39] who found that sodium glucan sulfate induced ulcerative colitis through PI3K/AKT/NF-κB signaling pathway. *VTN*, an adhesive multifunctional glycoprotein, stimulated tissue repair and regeneration and promoted cell adhesion, migration and degradation of the matrix [40]. The NF-κB signaling pathway was a positive regulator of *VTN* expression [40], hense, these results suggested that activation of the NF-κB pathway led to higher expression of *VTN*. *SPP1* induces a proinflammatory microenvironment by recruiting leukocytes to sites of inflammation, regulating the production of cytokines and cell activation, and inhibiting lymphocyte apoptosis [41]. *CYP2C18* is a metabolic enzyme-encoding gene that is up-regulated when the body is stimulated by inflammation or toxic substances [42]. The present study indicated that that CN-LPS group induced salpingitis by up-regulating the expression of *VTN*, *SPP1* and *CYP2C18*. *MOGAT1*, a gene involved in lipid metabolism, is upregulated leading to fatty liver [43]. *HK2* has been linked to malignant growth in many types of cancer, where it plays a key role in aerobic glycolysis [44]. *CA4* plays a crucial role in maintaining acid–base balance in the internal environment and assists MCT4 in transporting *L*-lactic acid outside the cell, accelerating acidification in the external environment [45]. However, downregulation of *CA4* is mainly associated with tumors and cancers, such as colon cancer [46]. With down-regulation of *CNTN3*, *EGFR* may be abnormally activated and initiate ErbB signaling to promote tumor growth and invasion [47]. *ACAN* is a major component of the extracellular matrix. Excessive production of pro-inflammatory factors led to increased production of matrix metalloproteinases (MMPs), other cytokines and prostaglandins, which would lead to decreased synthesis of *ACAN* and ultimately degradation of the extracellular matrix [48]. In this study, *HK2* and *MOGAT1* were upregulated and *CA4*, *CNTN3,* and *ACAN* were downregulated in the CN-LPS group, suggesting that LPS-induced salpingitis caused abnormal lipid and sugar metabolism and altered the acid–base balance of the internal environment, degrading the extracellular matrix. Dietary supplementation with MLP inversely modulated the above genes, suggesting that MLP was effective in suppressing salpingitis induced by LPS. The oviduct is the main reproductive organ of laying hens, and the induction of salpingitis directly affects the reproductive performance of laying hens. *ESR1* is a ligand-dependent nuclear receptor, which is closely related to the reproductive performance of laying hens. It had been shown that chickens that were deprived of estrogen and nuclear receptors had reproductive defects, such as fewer follicles, slower ovarian follicle growth, and a reduced ability to ovulate [49]. In this study, *ESR1* expression was highly modulated in the MLP-LPS group compared to the CN-LPS group, suggesting that MLP mitigates the damage of salpingitis on the reproductive performance of laying hens through high expression of *ESR1*. The uterus serves as the primary site for eggshell formation; thus the regulation of calcium was crucial in the uterus. The calcium-related genes involved in this study are *KLHL1*, *CPNE4*, *CHGA*. *KLHL1* interacted with channel proteins to regulate the function of calcium ion channels, and its deletion resulted in different changes in calcium current and neuronal excitability [50]. *CPNE4*, a gene that encodes a calcium-dependent phospholipid binding protein, is suppressed in wound infections [51]. *CHGA* is a signaling factor secreted by chromaffin granules of neurosecretory cells and involved in Ca^2+^ and catecholamine metabolism [52]. *CHGA* indirectly promotes the development of intestinal inflammation by modulating epithelial repair through selective activation of macrophages [53]. The results of our study indicated that CN-LPS group affected calcium ion regulation by up-regulating *CHGA* and inhibiting the expression of *KLHL1* and *CPNE4*. This suggested that salpingitis might lead to abnormal eggshell formation by affecting the expression of genes involved in calcium ion regulation. The effect of salpingitis on eggshells in the MLP-LPS group might be mitigated by down-regulating *CHGA* and up-regulating *KLHL1* and *CNPE4*. In summary, *Lactiplantibacillus plantarum* prevented salpingitis by inhibiting the expression of inflammatory genes, promoting the expression of anti-tumor genes, and increasing the expression of calcium-regulated genes. The effect of MLP on the expression of related genes was likely due to promoting the growth of beneficial bacteria and inhibiting the overgrowth of harmful bacteria [3, 54].

The gut microbiota played a key role in intestinal ecology [55]. This study indicated that dietary supplementation with MLP increased the abundance of Firmicutes, which were involved in the digestion of various nutrients and improved host health by producing SCFAs, inhibiting inflammation [56, 57]. Due to the increase of Firmicutes, the immune response was weakened and the intestinal barrier function was enhanced [58]. By analyzing the composition of gut microbiota at the genus level, the hens in CN-LPS had higher levels of *Bacteroides* and *Rikenellaceae_RC9_gut_group*, while *Ruminococcus_torques_group* and *Phascolarctobacterium* were lower than those in the CN and MLP-LPS groups. The abundances of *Lactobacillus*, *Faecalibacterium* and *Megamonas* in MLP-LPS group were higher than those from other groups. Additionally, LEfSe analysis revealed that the most abundant discriminant taxon was *Candidatus-Saccharimonas* in the CN-LPS group and *Rhodococcus* in the MLP-LPS group. The benefits of *Lactobacillus* included maintaining intestinal microbiota, improving immunity, regulating cholesterol metabolism, and promoting nutrient absorption [15, 59]. The bacterium *Faecalibacterium* could produce large amounts of butyrate and anti-inflammatory factors, such as shikimic and IL-10, as well as inhibit the activation of NF-κB and the production of pro-inflammatory cytokines [60, 61], which also explained why MLP supplementation enhanced the plasma IL-10 level in the LPS-induced hens in this study. *Megamonas* induced regulatory T-cell differentiation by producing propionate to enhance host health [62]. Furthermore, *Rhodococcus* had a wide range of metabolic abilities, including the degradation of toxic chemicals and the synthesis of valuable compounds [63].The abundance of *Bacteroides* was positively correlated with the expression of pro-inflammatory cytokines, such as IL-1β and TNF-α, which were increased when the gut was pathologically damaged and promoted inflammation, which might disrupt the intestinal barrier [59, 64]. *Rikenellaceae RC9 gut group* was positively correlated with harmful bacteria, and its abundance increase might further induce dysbiosis [65]. The *Candidatus Saccharimonas* inhibited TNF production within macrophages and was associated with inflammatory mucosal diseases [66]. *Ruminococcus_torques_group* was closely associated with obesity and metabolic syndrome, and that it was beneficial for metabolism by increasing deoxycholic acid production and improving obesity through the bile-acid–adipose TGR5 axis [61]. *Phascolarctobacterium* reduced inflammation and protected the intestinal mucosal barrier by producing propionate [67]. In conclusion, *Bacteroides, Rikenellaceae_RC9_gut_group* and *Candidatus Saccharimonas* had been found to promote the proliferation of pathogenic bacteria by disrupting the intestinal barrier, which may lead to an increase in toxic metabolites production [68, 69]. Conversely, *Ruminococcus_torques_group* and *Phascolarctobacterium* had been shown to improve metabolism of fatty acids and bile acids and enhanced the integrity of the intestinal barrier [61, 67]. This explained why the upregulation of beneficial bacteria, such as *Phascolarctobacterium*, relieved the inflammatory response and intestinal barrier damage caused by LPS challenge.

**Fig. 7:**
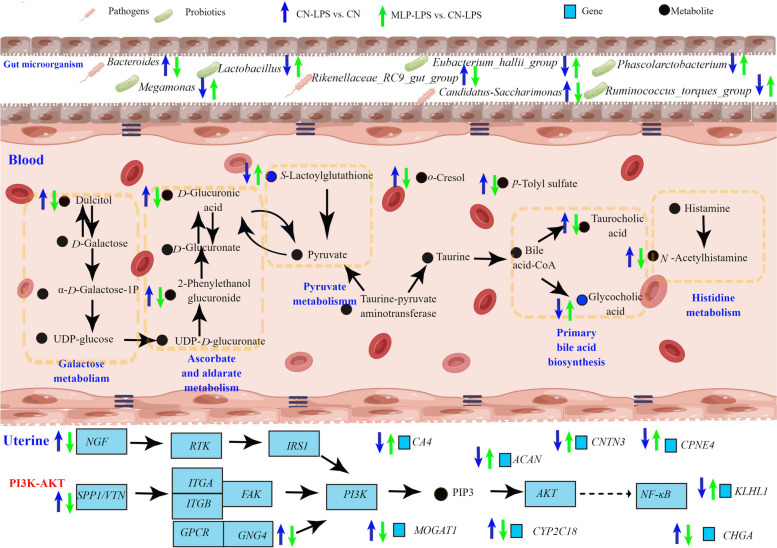
Schematic diagram of regulatory network of MLP for prevention of salpingitis. Arrows beside microbes, genes, and metabolite names indicate increases (upward) or decreases (downward) in microbial abundance, gene expression, and metabolite content. Blue arrows indicate CN-LPS vs. CN and green arrows indicate MLP-LPS vs. CN-LPS. The group after ‘vs.’ represents the control group

Recently, it was shown that *Lactobacillus plantum* GIM17 improved colonic inflammation by increasing the relative abundance of beneficial bacteria (*Actinomycetes*) and raising the levels of beneficial serum metabolites [70]. In addition, altered blood metabolites, such as SCFAs, derived from changed gut microbiota could ameliorate apoptosis of ovarian granulosa cells [71]. To investigate the connection between the gut microbiome and distal uterine inflammation, we performed an analysis of plasma metabolite features using untargeted metabolite profiles and demonstrated significantly different plasma metabolite profiles in the CN, CN-LPS, and MLP-LPS groups. In the present study, LysoPA (24:0/0:0), *p*-tolyl sulfate, 2-phenylethanol glucuronide, *p*-cresol glucuronide, *N*-acetylhistamine, dulcitol and taurocholic acid were significantly higher in CN-LPS group, while *S*-lactoylglutathione and glycocholic acid were significantly lower expressed. LysoPA (24:0/0:0) was a lysophosphatidic acid. Rancoule et al. [72] stated that lysophosphatidic acid (LPA) was highly expressed in pathological inflammation, which was consistent with the results in this study. The high expression of LysoPA (24:0/0:0) was due to increased pro-inflammatory factors (TNF) induced by LPS, which produced a large amount of lysophospholipase D autotaxin (ATX), and ATX produced LPA [72]. Dietary supplementation with MLP reduced the level of LysoPA (24:0/0:0), presumably because of reducing the level of pro-inflammatory cytokines. *p*-Tolyl sulfate and *p*-cresol glucuronide were generated by phenylalanine and tyrosine decomposition with anaerobic bacteria, which promoted the expressions of inflammatory genes, such as interleukin-6 (IL-6) [73, 74]. This explained why elevated levels of *p*-tolyl sulfate and *p*-cresol glucuronide were associated with increased inflammation in the CN-LPS group. In pyruvate metabolism, *S*-lactoylglutathione could be hydrolyzed into glutathione by hydroxyacylglutathione hydrolase [75]. In this study, the CN-LPS group might reduce the amount of glutathione by lowering *S*-lactoylglutathione, which led to increased inflammation. In CN-LPS group, high expression of *N*-acetylhistamine, a metabolite of histidine, led to abnormal histidine metabolism, which in turn impinged on histidine's anti-inflammatory effects [76]. 2-Phenylethanol glucuronide and dulcitol are toxic products produced by the abnormal metabolism of uronic acid and galactose, and a significant increase in the concentration of the former may result in a lack of glucose, and a high expression of the latter can trigger inflammation [77, 78]. Li et al. [79] showed that *L. plantarum* significantly reduced the relative content of 2-phenylethanol glucuronide, suggesting that *L. plantarum* NCU116 has a potential effect on improving metabolism of uronic acid, which was consistent with the present study. In addition, MLP altered the metabolism of primary bile acids, indicating a decrease in taurocholic acid and an increase in glycocholic acid. A previous study had pointed out that high levels of taurocholic acid caused epithelial paracellular permeability and barrier dysfunction of the ileum [80]. Taken together, MLP group alleviated salpingitis by altering the composition of beneficial and harmful bacteria, downregulating galactose, uronic acid, and histidine, upregulating pyruvate, and improving primary bile acid metabolism, and then inversely regulating the expressions of LPS-induced genes involved in inflammation, reproduction, and calcium ion transport (Fig. 7).

The effect of *Lactiplantibacillus plantarum* on salpingitis was further elucidated by analyzing the correlation of differential metabolites, microorganisms and genes. Based on the results of the correlation analysis, it was observed that the levels of *Phascolarctobacterium*, *Eubacterium_hallii_group*, and *Ruminococcus_torques_group* were elevated, while the levels of *Candidatus_Saccharimonas* and *Rikenellaceae_RC9_gut_group* were reduced by MLP supplementation. These changes in the microbial composition contributed to the improvement of the intestinal barrier and the reduction of inflammation [65–67, 81]. The intestinal barrier was improved to reduce toxic substances in the plasma, which explained the reduced levels of *N*-acetylhistamine, *p*-tolyl sulfate, and *o*-cresol in the plasma [68, 69]. Subsequently, changes in these metabolites resulted in elevated levels of the genes *ACAN*, *CNTN3* and *CPNE* and decreased levels of *VTN*, *CYP2C18* and *MOGAT1*. Changes in these genes alleviated inflammation and cytoplasmic degradation and improved metabolism [40, 42, 43, 47, 48] (Fig. 6C).

## Conclusions

In summary, the molecular regulatory network of microencapsulated *Lactiplantibacillus plantarum* for prevention of salpingitis is to reduce the abundance of harmful bacteria *Candidatus_Saccharimonas* and increase the abundance of beneficial bacteria *Phascolarctobacterium, Ruminococcus_torques_group* and *Eubacterium_hallii_group*, which could increase the content of beneficial metabolites *S*-lactoylglutathione and reduce the content of toxic metabolites *p*-toyl sulfate, *o*-cresol, and *N*-acetylhistamine. Ultimately, it promoted the expression of anti-tumor genes, increased the expression of calcium-regulated genes, and reduced the degradation of extracellular matrix. This study provides valuable information for studying the prevention of salpingitis by *Lactiplantibacillus plantarum*.

### Supplementary Information

**Additional file 1: Table S1. **Ingredients and nutrient composition of the basal diet.** Table S2. **Sequences of real-time PCR primers.** Table S3. **Effect of dietary MLP on cecal microflora diversity in laying hens challenged with LPS.** Fig. S1. **Validation of relative expression levels of transcriptome candidate genes. 

## Data Availability

Datasets used or analyzed in this study are available by reasonable request from the corresponding author.

## Abbreviations

ACAN: Aggrecan
ASIC4: Acid sensing ion channel subunit family member 4
BIRC5: Baculoviral IAP repeat containing 5
CA4: Carbonic anhydrase 4
CHAC1: ChaC glutathione specific gamma-glutamylcyclotransferase 1
CHGA: Chromogranin A
CISH: Cytokine inducible SH2 containing protein
CNTF: Ciliary neurotrophic factor
CNTN3: Contactin 3
COX2: Cytochrome c oxidase subunit II
CPNE4: Copine 4
CYP2C18: Cytochrome P450 family 2 subfamily C member 18
ELISA: Enzyme-linked immunosorbent assay
ESR1: Estrogen receptor 1
FGF7: Fibroblast growth factor 7
GNG4: G protein subunit gamma 4
HK2: Hexokinase 2
IL-10: Interleukin-10
IL18BP: Interleukin 18 binding protein
IL-1β: Interleukin-1β
IL-2: Interleukin-2
IL-6: Interleukin-6
INF-γ: Interferon-γ
INOS: Nitric oxide synthase 2
KLHL1: Kelch like family member 1
MOGAT1: Monoacylglycerol O-acyltransferase 1
MYD88: Myeloid differentiation primary response 88
NF-κB: Nuclear factor kappa B
NGF: Nerve growth factor
RPS6KA2: Ribosomal protein S6 kinase A2
SOCS1: Suppressor of cytokine signaling 1
SPP1: Secreted phosphoprotein 1
SRM: Spermidine synthase
TLR2: Toll like receptor 2
TLR4: Toll like receptor 4
TNF-α: Tumor necrosis factor alpha
VTN: Vitronectin
