# Supplementary material for: Multi-omics analysis reveals the molecular regulatory network underlying the prevention of Lactiplantibacillus plantarum against LPS-induced salpingitis in laying hens
Source: J Anim Sci Biotechnol. 2023 Nov 17;14:147. doi: 10.1186/s40104-023-00937-x (PMC10655300; doi:10.1186/s40104-023-00937-x)
Supplement: Supplementary file 1 — Additional file 1: Table S1. Ingredients and nutrient composition of the basal diet. Table S2. Sequences of real-time PCR primers. Table S3. Effect of dietary MLP on cecal microflora diversity in laying hens challenged with LPS. Fig. S1. Validation of relative expression levels of transcriptome candidate genes. [file 40104_2023_937_MOESM1_ESM.docx]

**Table S1** Ingredients and nutrient composition of the basal diet. (air-dry basis)

| Ingredient | Composition (%) |
| --- | --- |
| Corn | 61.00 |
| Soybean meal | 25.58 |
| Limestone | 9.00 |
| Dicalcium phosphate | 1.90 |
| Soybean oil | 1.80 |
| Vitamin premix^a^ | 0.02 |
| Minerals premix^b^ | 0.20 |
| Choline chloride (50%) | 0.20 |
| Salt | 0.15 |
| L-Methionine | 0.10 |
| Lysine | 0.05 |
| Total | 100.00 |
| Calculated chemical composition (%) |  |
| ME (MJ/kg) | 11.41 |
| Crude protein | 16.50 |
| Calcium | 3.64 |
| Total Phosphorus | 0.63 |
| Lysine | 0.81 |
| Methionine | 0.35 |

^a^Vitamin premix provided the following per kilogram of diet: vitamin A, 12,500 IU; vitamin D_3_, 2500 IU; vitamin E, 15 IU; vitamin K_3_, 2.65 mg; vitamin B_1_, 2 mg; vitamin B_2_, 6 mg; vitamin B_12_, 0.025 mg; nicotinic acid, 50 mg; calcium pantothenate, 12 mg; biotin, 0.0325 mg; folic acid, 1.25 mg.

^b^The mineral premix provided the following per kg of diet: iron, 80 mg; copper, 8 mg; manganese, 100 mg; zinc, 75 mg; iodine, 0.35 mg; selenium, 0.15 mg.

Table S2 Sequences of real-time PCR primers.

| Gene name | Primer (5′—3′) | GenBank ID | Products |
| --- | --- | --- | --- |
| *TLR4* | F：ACGGAAGGCTTTGGTTGGGATT | NM_001030693.1 | 184 |
|  | R：GATGTTGCTATCTGGTGCTTGGAA |  |  |
| *TLR2* | F：CCTGGTGTTCCTGTTCATCCTCAT | NM_001161650.2 | 173 |
|  | R：AGTTGGAGTCGTTCTCACTGTAGG |  |  |
| *MYD88* | F：TCTGGTGACTGTGGAGCAAGGAA | NM_001030962.4 | 206 |
|  | R：CCGCTTGTAGGAAGGCACTAATGG |  |  |
| *NF-κB* | F：CCACAACACAATGCGCTCTG | NM_205129.1 | 112 |
|  | R：AACTCAGCGGCGTCGATG |  |  |
| β-actin | F：ATTGTCCACCGCAAATGCTTC | NM_205518.1 | 113 |
|  | R：AAATAAAGCCATGCCAATCTCGTC |  |  |
| *TNF-α* | F：AGTGCTGTTCTATGACCGCC | NM_204267.1 | 88 |
|  | R：CGCTCCTGACTCATAGCAGA |  |  |
| *COX-2* | F：TGTCCTTTCACTGCTTTCCAT | NM_001167719.1 | 84 |
|  | R：TTCCATTGCTGTGTTTGAGGT |  |  |
| *IL-1β* | F：ATCGTTTATGTTTCATTACCGTCC | XM_015297469.3 | 122 |
|  | R：CTCCAGTCACAATAAATACCTCCAC |  |  |
| *INOS* | F：CCTGGAGGTCCTGGAAGAGT | NM_204961.2 | 82 |
|  | R：CCTGGGTTTCAGAAGTGGC |  |  |
| *SPP1* | F：GCCCAACATCAGAGCGTAGA | NM_204535.5 | 204 |
|  | R：ACGGGTGACCTCGTTGTTTT |  |  |
| *HK2* | F：TGGAGGTGAAGCGGAGGATGAG | XM_046931558.1 | 177 |
|  | R：GCACCAGCAGCACACGGAAG |  |  |
| *CA4* | F：GGAAGCAAACAGTCACCCATC | XM_040687663.2 | 224 |
|  | R：ACTCCCCAGTGCAGATGAAA |  |  |
| *SOCS1* | F：CTACTGGGGACCGCTGACC | XM_046927473.1 | 117 |
|  | R：TTAACACTGATGGCAAAGAAACAA |  |  |

Table S3 Effect of dietary MLP on cecal microflora diversity in laying hens challenged with LPS.

| Item | CN | CN-LPS | MLP-LPS | SEM^a^ | *P*-value |
| --- | --- | --- | --- | --- | --- |
| shannon | 4.76 | 4.65 | 4.63 | 0.041 | 0.362 |
| simpson | 0.02 | 0.02 | 0.02 | 0.001 | 0.783 |
| ace | 712.24 | 724.69 | 705.51 | 10.887 | 0.792 |
| chao | 712.72 | 736.47 | 719.63 | 13.190 | 0.779 |

^a^Each mean represents 6 replicate cages; SEM, standard error of the mean.

CN, Basal diet; CN-LPS, Basal diet+LPS; MLP-LPS, Basal diet+MLP+LPS.


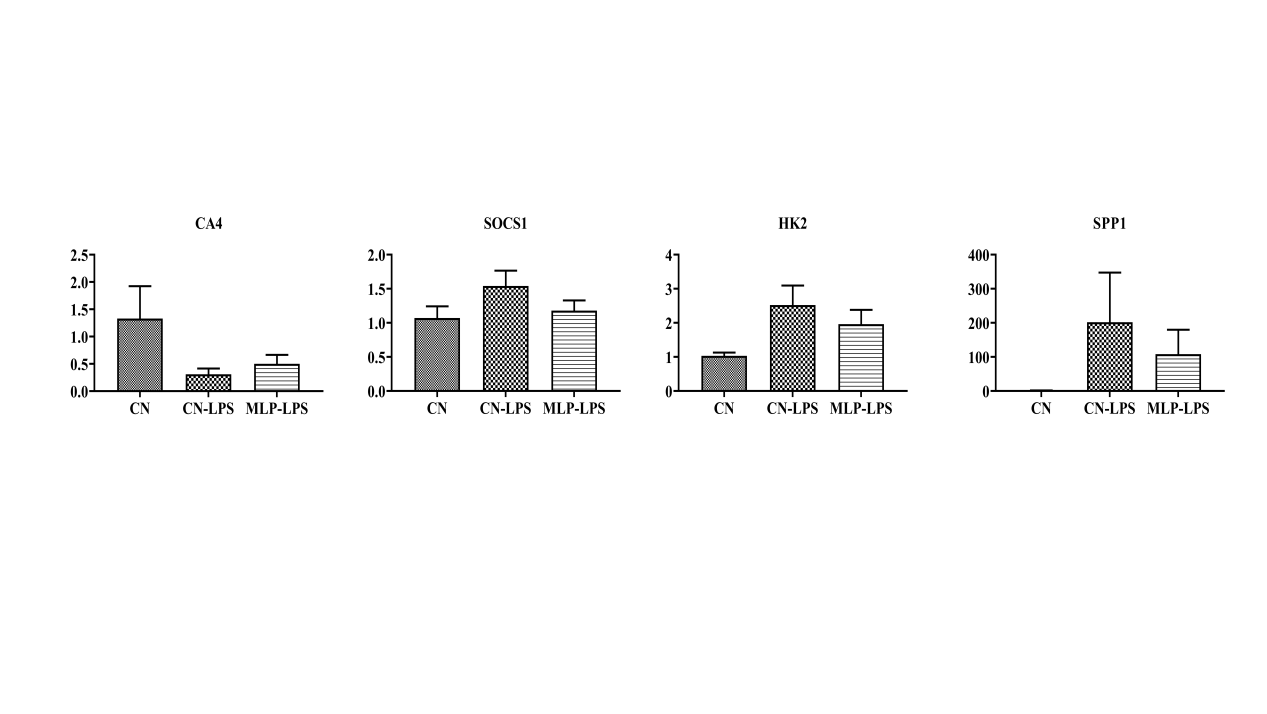


Fig. S1. Validation of relative expression levels of transcriptome candidate genes. Values were means and standard errors, CN, Basal diet; CN-LPS, Basal diet+LPS; MLP-LPS, Basal diet+MLP+LPS.
